# Neovasculature can be induced by patching an arterial graft into a vein: A novel *in vivo* model of spontaneous arteriovenous fistula formation

**DOI:** 10.1038/s41598-018-21535-2

**Published:** 2018-02-16

**Authors:** Yukinobu Ito, Makoto Yoshida, Daichi Maeda, Masato Takahashi, Hiroshi Nanjo, Hirotake Masuda, Akiteru Goto

**Affiliations:** 1 Department of Cellular and Organ Pathology, Graduate School of Medicine, Akita University, Akita, Japan; 2 Department of Diagnostic Pathology, Akita Kousei Medical Center, Akita, Japan; 3 Department of Clinical Pathology, Akita University Hospital, Akita, Japan; 4 Department of Clinical Laboratory, Ogachi Central Hospital, Akita, Japan

## Abstract

Arteriovenous malformations consist of tangles of arteries and veins that are often connected by a fistula. The causes and mechanisms of these clinical entities are not fully understood. We discovered that suturing an arterial patch into the common jugular vein of rabbits led to spontaneous neovascularization, the formation of an arteriovenous fistula and the development of an arteriovenous shunt. An arterial patch excised from the common carotid artery was sutured into the common jugular vein. Within a month, a dense nidus-like neovasculature formed around the patch. Angiography and pulse-oximeter analyses showed that the blood flowing into the neovasculature was arterial blood. This indicated that an arteriovenous shunt had formed. Fluorescence *in situ* hybridization with a Y chromosome probe in female rabbits that received an arterial patch from male rabbits showed that the vessels close to the graft bore the Y chromosome, whereas the vessels further away did not. Enzyme-linked immunosorbent assays and cDNA microarray analysis showed that multiple angiogenic factors were upregulated after patch transplantation. This is the first *in vivo* model of spontaneous arteriovenous fistula formation. Further research on these differences may help to improve understanding of human vascular anomaly diseases and the basic principles underlying vasculogenesis and/or angiogenesis.

## Introduction

Arteriovenous malformations (AVMs) are generally defined as vascular anomalies in which blood flows directly from an artery into a vein: the capillary network is bypassed. AVMs can occur in various organs in the human body, including the brain, lungs, liver, kidneys, uterus, intestinal tract, spinal dura and placenta (chorangioma)^1–6^. AVMs usually consist of a feeder artery, a drainage vein, and a tangle of arteries and veins that are connected by one or more fistulae. AVMs can be asymptomatic or they can present with life-threatening symptoms, including stroke, brain abscess, haemorrhage and hypoxaemia. The exact causes of AVMs and their pathogenesis are not yet understood. In particular, the process of arteriovenous fistula (AVF) formation, which is a crucial step in AVM pathogenesis, is not clear. Moreover, it is not yet known whether and how angiogenic factors, including vascular endothelial growth factor A (VEGFA), are involved in AVM formation.

The paucity of knowledge about the chronological processes that participate in the formation of AVM/AVF reflects in part the fact that early lesions are very rarely detected in clinical practice and in part the lack of suitable animal models of AVM pathogenesis. The lack of animal models in particular has slowed the development of therapeutic medications for AVMs^7^. While a mouse brain AVM model was developed recently, it was generated by genetic engineering: the activin receptor-like kinase-1 (ALK-1) gene was deleted using the SM22-Cre transgene^7^. It is uncertain whether this model reflects the natural pathogenesis of AVMs/AVFs. Models of spontaneous AVF formation that do not involve genetic engineering have not yet been reported.

In the present study, we developed a novel *in vivo* animal model of neovasculature induction and spontaneous AVF formation. This model was the result of a fortuitous finding that arose during our studies on the differences between arteries and veins in rabbits: we discovered that, when we sutured an arterial graft patch into a rabbit vein, an AVM formed. The methods used to produce the model are described here. In addition, we performed histological, haemodynamic and molecular biological experiments to elucidate the chronological processes that are involved in the development of this neovasculature. These data are described here.

## Results

### Time course of growth of new blood vessels after grafting an arterial patch into a vein

#### Macroscopic observations

**Figure 1:**
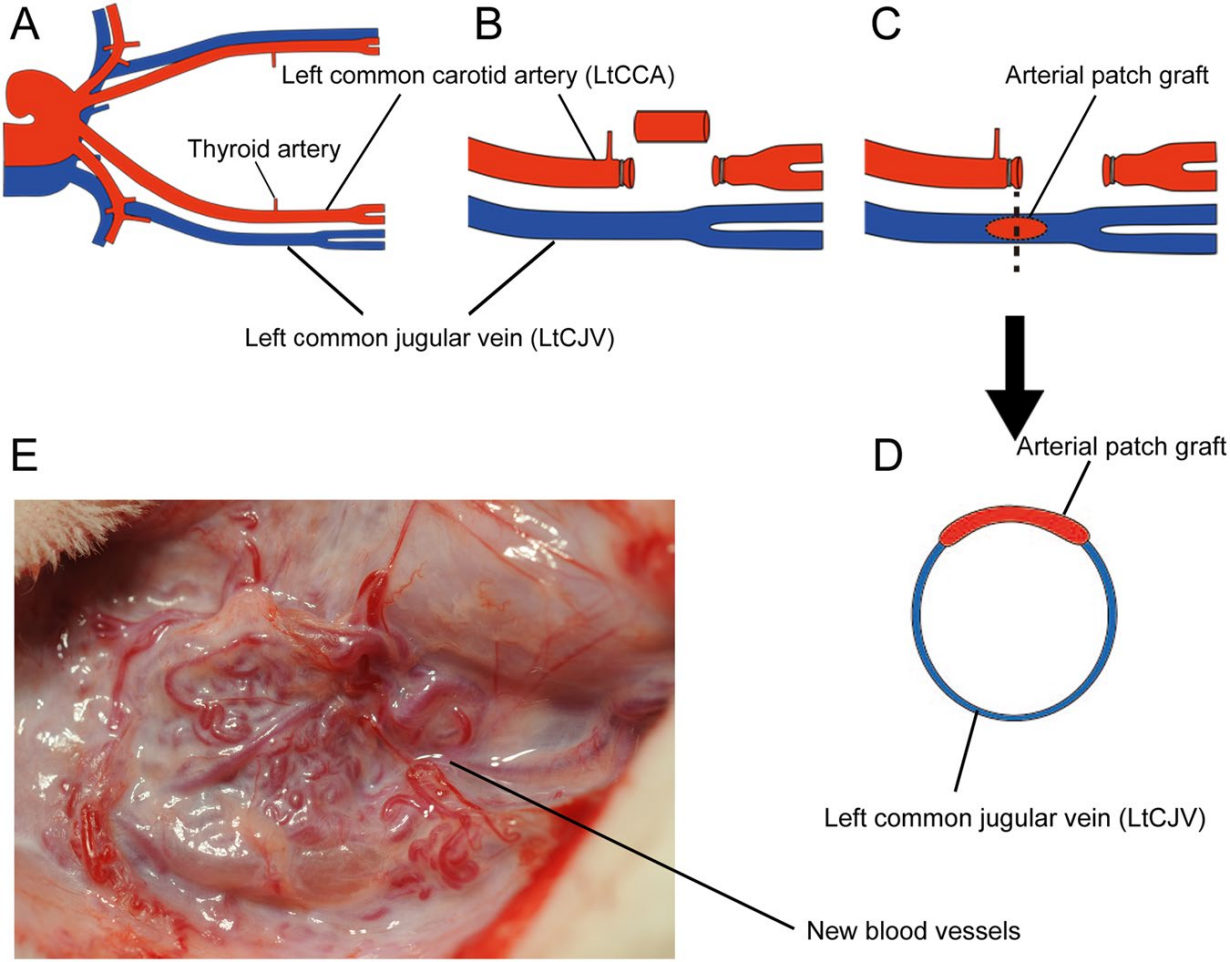
The patch procedure used to generate the spontaneous arteriovenous fistula formation model. (**A**) The anatomy of the cervical vessels in rabbits. (**B**) The patch procedure starts with dissection and ligation of the left common carotid artery (LtCCA) about 10 mm superior to the thyroid artery. The artery is cut so that 6–7 mm of arterial tissue can be collected. The arterial tissue is opened up into a rectangle and then trimmed to form an oval. Thereafter, the left common jugular vein (LtCJV) is revealed by dissection and a 7 mm incision is made into the blocked portion under haemostasis. (**C**) The trimmed arterial tissue is sewn into the LtCJV in a patchwork manner. (**D**) A transverse section of the LtCJV bearing the arterial patch is shown. (**E**) One year after the patch procedure, neovasculature was induced around the arterial patch graft. The neovasculature resembled arteriovenous malformation (AVM).

The left common carotid artery (LtCCA) was disconnected and arterial tissue with a length of 6–7 mm was collected (Fig. 1). The disconnected arterial graft was opened into a rectangular shape (5 × 4 mm), trimmed into an oval shape and sutured to the left common jugular vein (LtCJV) using a patchwork technique and 8–0 nylon. This protocol is hereafter called the “patch” procedure.

**Figure 2:**
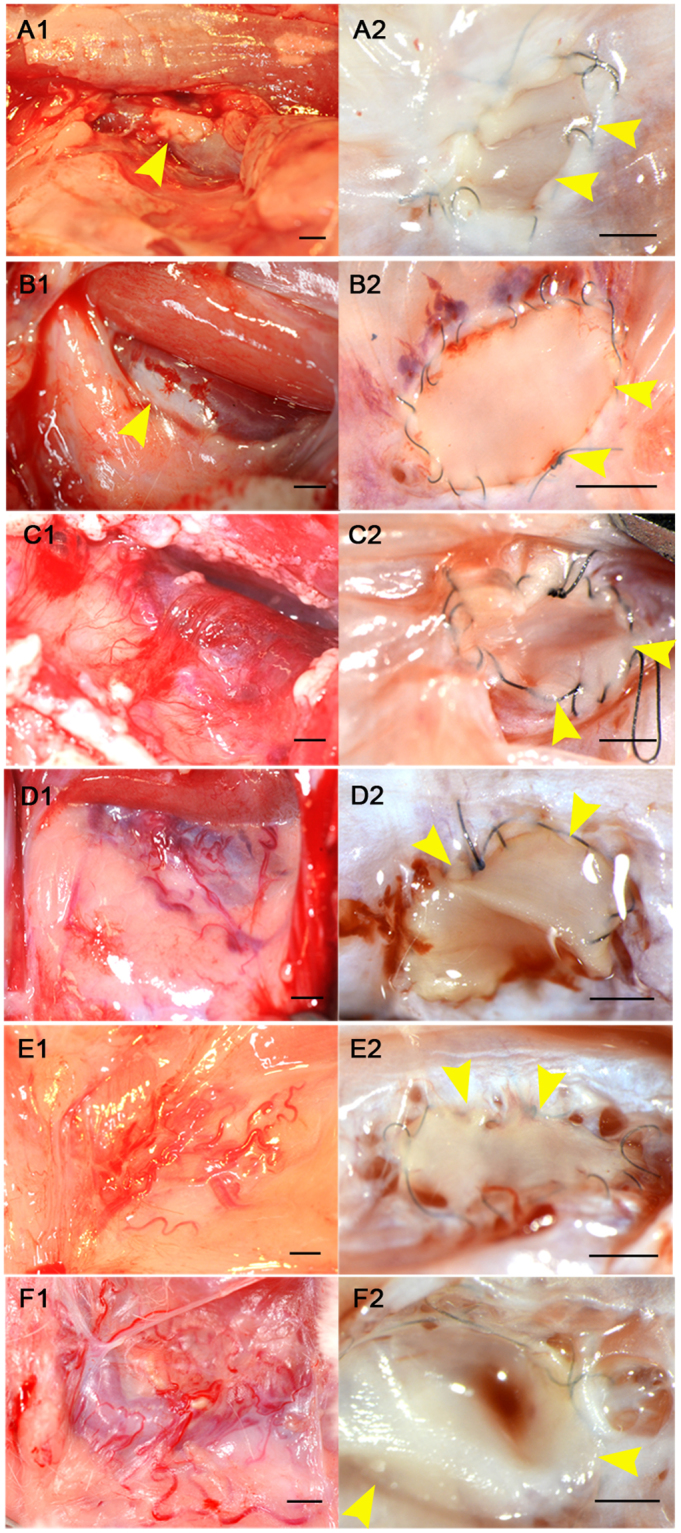
Formation of new blood vessels over time. (**A1**–**F1**) are representative macroscopic views of the patch-bearing vein on days 3, 7, 10, 14, 28 and 84, respectively. (**A2**–**F2**) are representative stereomicroscope photographs of the luminal side of the arterial patch (yellow arrowhead) on the vein on the same days. On day 3, blood vessels and the apertures of new blood vessels around the arterial tissue (yellow arrowhead) cannot be seen (**A1** and **A2**). On day 7, there are hazy capillary-like structures around the adipose tissue but no holes (**B1** and **B2**). On day 10, the LtCJV turned a brighter red colour and there were a few small holes around the margin of the arterial patch (**C1** and **C2**). On day 14, the adipose tissue around the arterial patch had clearly visible neovasculature that flexed and meandered and the holes had become larger (**D1** and **D2**). On day 28, the new blood vessels and the shunting were even more pronounced and the apertures of the new blood vessels at the margin of the patch were even larger (**E1** and **E2**). On day 84, there were many new blood vessels around the common jugular vein and in its surrounding adipose tissue and the vein was brighter than before and had turbulent blood flow (**F1**). There were large holes around the arterial patch (**F2**). Scale bar = 5 mm.

Macroscopic observations over 84 days showed that, at day 3, there were no new blood vessels growing from the patched vessel (Fig. 2A1). However, on day 7, there were hazy capillary-like structures around the adipose tissue (Fig. 2B1). On day 10, the LtCJV turned a brighter red colour, which suggested that arterial blood was flowing into the LtCJV (Fig. 2C1). On day 14, the adipose tissue around the arterial graft had neovasculature that was readily macroscopically visible (Fig. 2D1). The new blood vessels could be distinguished from the pre-existing linear blood vessels because they exhibited strong flexion and significant meandering. The inflow of arterial blood was even more prominent on day 14. Turbulence in the LtCJV was also observed at this point. On day 28, the new blood vessels and the shunting were even more pronounced (Fig. 2E1). On day 84, there were many new blood vessels around the common jugular vein and in its surrounding adipose tissue (Fig. 2F1).

Stereoscopic microscopy of the lumen of the transplanted LtCJV showed that, on days 3 and 7, there were no holes in the vessel wall: these holes signify the presence of blood vessels arising from the vessel (Fig. 2A2,B2). However, on day 10, there were tiny holes (around 1 mm in diameter) around the margin of the transplanted arterial tissue (Fig. 2C2). By day 14, the holes had become larger (Fig. 2D2). On day 28, the holes were larger still. Moreover, they were arranged around the arterial tissue such that the patch resembled a punch-out section (Fig. 2E2). On day 84, there were large holes around the arterial patch (Fig. 2F2).

#### Histology

**Figure 3:**
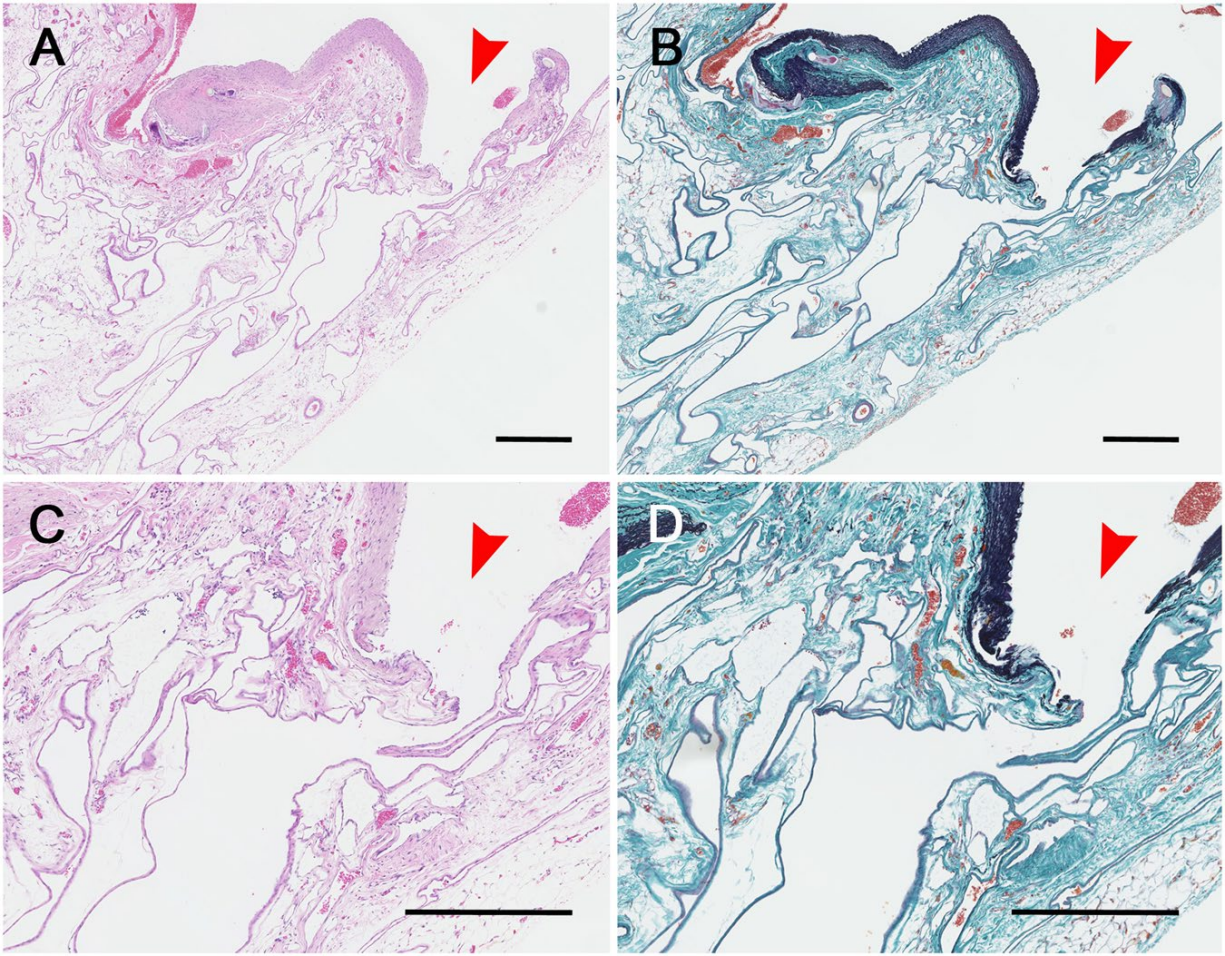
Histological findings 28 days after the patch procedure. (**A** and **B**) Histology of the arterial patch on the left common jugular vein on day 28 showed that new blood vessels had opened at the margin of the arterial patch (**A**: Haematoxylin-Eosin-stain, 40×; **B**: Elastica-Masson-stain, 40×). (**C** and **D**) The new blood vessels had thin walls and extended in an irregular fashion (**C**: Haematoxylin-Eosin-stain, 150×; **D**: Elastica-Masson-stain, 150×). Scale bar for (**A–D**) = 0.5 mm. The red arrowhead indicates the opening of a new blood vessel.

Although there were no macroscopic changes in or around the arterial graft on day 3, histological analysis showed the presence of a dilated capillary-like structure in the adipose tissue at this time point. This structure was filled with red blood cells (data not shown). On day 7, the diameters of these structures had increased but they were fewer in number. The hazy capillary-like vessels, which were macroscopically visible at this time point, had extremely thin walls that contained poor-quality elastic fibres (data not shown). On day 10, there was a large number of tiny new blood vessels around the arterial graft. The diameters of these new vessels were larger than those observed on day 7. However, their number was almost identical. These vessels were irregularly dilated and had less elastic and collagen fibres than those observed earlier (data not shown). On day 28, there was neovasculature that varied in size and degree of dilation; the capillary-like vessels that were observed previously were no longer present (Fig. 3). There was no infiltration of inflammatory cells or granulation tissue around the arterial transplant at any time.

Of the 12 animals with the patch that were examined by histology on days 28, 56 and 84 and after 1 year, 10 (83.3%) had grossly apparent new blood vessel formation. This shows that the model is highly reliable. The two rabbits that did not exhibit new blood vessel formation only had fibrous granulation tissue formation on the luminal side of the arterial graft.

None of the three surgical control models (Supplementary Figure 1) exhibited neovasculature formation. Angiogenesis was also not observed in the venous patch-into-vein, venous patch-into-artery and arterial patch-into-artery models (Supplementary Figure 1).

### Functional demonstration of arteriovenous shunt formation

**Figure 4:**
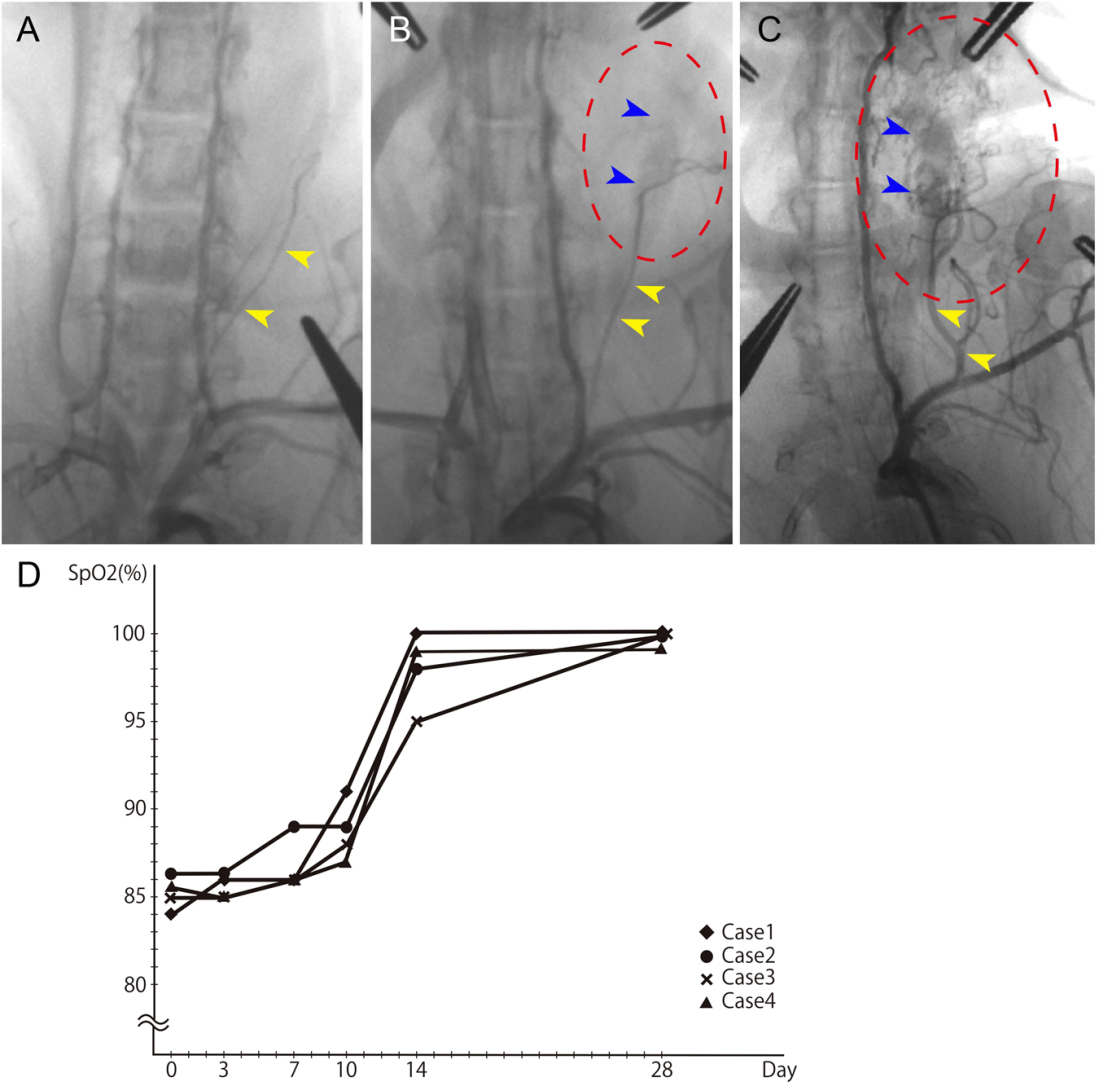
Formation of an arteriovenous shunt around the arterial patch, as shown by angiography and pulse-oximetry. Angiography of the arterial patch-bearing left common jugular vein was performed on day 3 (**A**) and day 28 (**B**) with contrast agent in the early arterial phase. The arteriovenous shunt had not formed on day 3 (yellow arrowheads, the left subclavian artery). On day 28, an arteriovenous shunt had formed, and its blood flow originated from the second branch of the left subclavian artery (yellow arrowheads). The left common jugular vein, which was a drainage vein, was enhanced in the arterial phase (blue arrowheads). (**C**) Angiography on day 84 showed that the feeding artery of the arteriovenous shunt had grown and that the new blood vessels were larger than they had been on day 28. The ovals in B and C that are demarcated by red dashes contain new blood vessels. Representative images are shown in (**A–C**). (**D**) Oxygen saturation in the left common jugular vein over time. Between day 0 and day 7, the oxygen saturation did not change. However, on days 10–14, the oxygen saturation started to rise sharply. On day 14 and thereafter, the oxygen saturation exceeded 95%. Thus, the blood in the vein after day 14 was of arterial origin, which indicated that the shunt was established around that time point. Scale bar = 1 cm.

Angiography was used to measure the *in vivo* blood flow in the neovasculature at various time points in the 84 days after surgery. The angiograms showed that blood from the second branch (ascending cervical artery) of the left subclavian artery (LtSA) was flowing into the new blood vessels and the LtCJV: this inflow was not observed on day 3 (Fig. 4A) and started as early as day 10 (data not shown). This suggests that placing the arterial patch into the LtCJV caused the formation of an arteriovenous shunt between the branch of the LtSA and the LtCJV that was mediated by the new blood vessels. The formation of the shunt started to be more readily recognizable on day 14 (data not shown). On day 28, it was possible to see the new blood vessels around the arterial tissue and adipose tissue on the angiogram (Fig. 4B). At that point, the shunt blood flow had increased and the new blood vessels and LtCJV came into sharper contrast. On day 84, angiography confirmed that arterial blood was flowing from the head side into the new blood vessels (Fig. 4C). Moreover, the feeder artery (*i*.*e*., the second branch of the LtSA) had become larger.

The pulse-oximetry measurements showed that the oxygen saturation of the LtCJV blood flow increased sharply between days 10 and 14 (Fig. 4D). This is consistent with the macroscopic, histological and angiographic observations and suggested that the AVF connecting the LtCJV to the LtSA occurred during days 10–14.

### Origin of the vascular network around the arterial patch, as assessed by fluorescence *in situ* hybridization (FISH)

**Figure 5:**
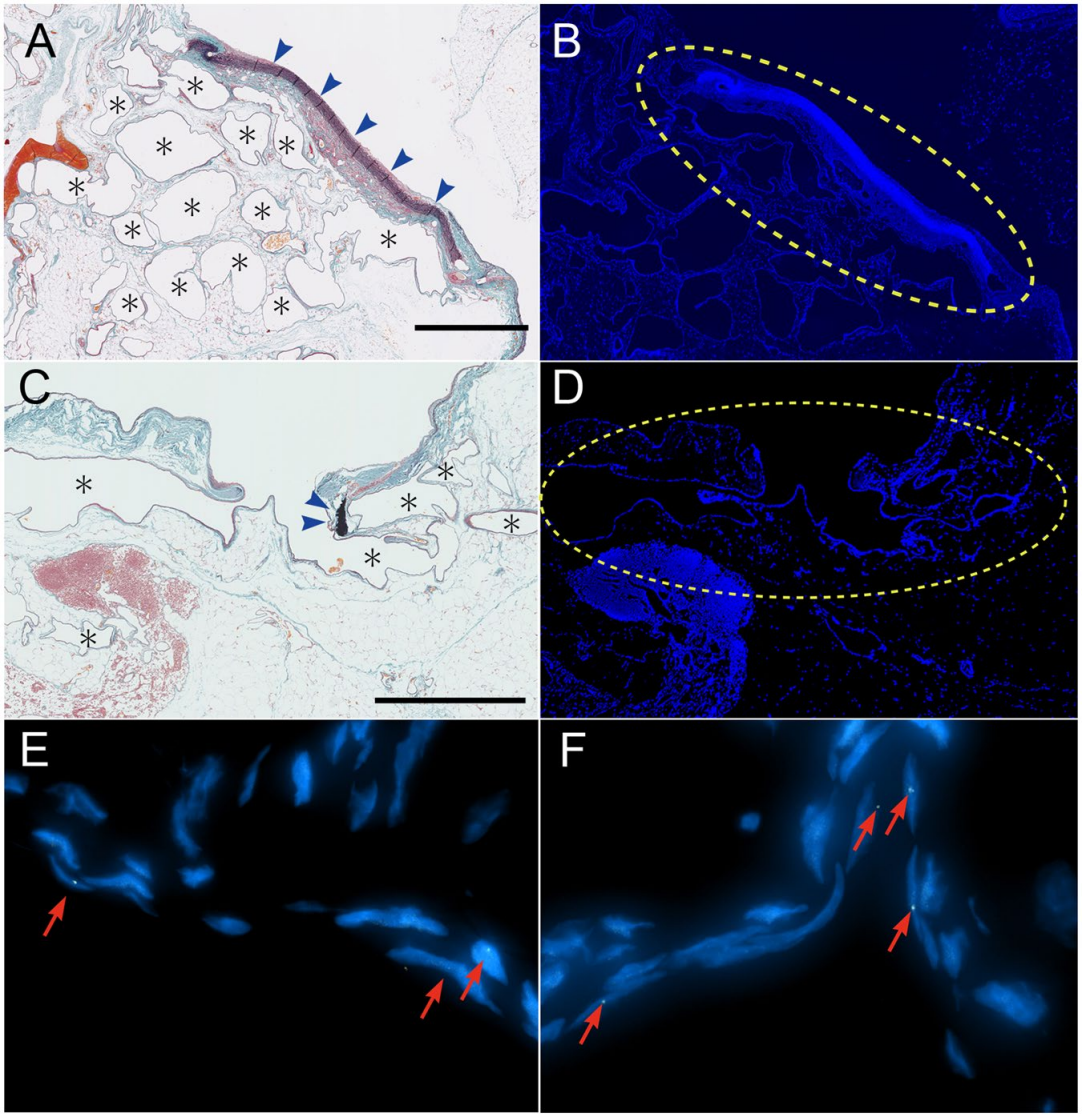
Origin of the new vessels, as shown by fluorescence *in situ* hybridization (FISH). The patch procedure was performed in two female rabbits with an arterial patch from male rabbits. FISH was performed on day 28 with a Cy3-labelled Y chromosome-specific probe. Representative images are shown. (**A** and **C**) Elastica-Masson-stained sections of the patch-bearing left common jugular vein. There were new blood vessels (indicated by *) around the arterial tissue (blue arrowhead) (magnification, 40×). (**B**–**F**) FISH of the same sections. The Y chromosome of the patch donor was detected in the area circled by the yellow dotted lines (**B** and **D**; magnification, 40×). The endothelial cells of the new blood vessels bore the Y chromosome-specific probe (red arrow) (**E** and **F**; magnification, 400×). Scale bar = 5 mm.

For this set of experiments, the LtCJV of female rabbits was transplanted with a patch from the LtCCA of male rabbits. Fluorescence *in situ* hybridization (FISH) using a rabbit Y chromosome-specific probe showed the presence of the yellow-coloured probe in the endothelial cells of the new blood vessels around the graft on day 28 (Fig. 5). However, the blood vessels in the adipose tissue that were more than 1 mm away from the arterial graft did not bear Y chromosome signals. Thus, the patch was the source of the new blood vessels.

### Changes in angiogenic factors during AVM formation, as determined by enzyme-linked immunosorbent assays (ELISAs) and cDNA microarray analyses

**Figure 6:**
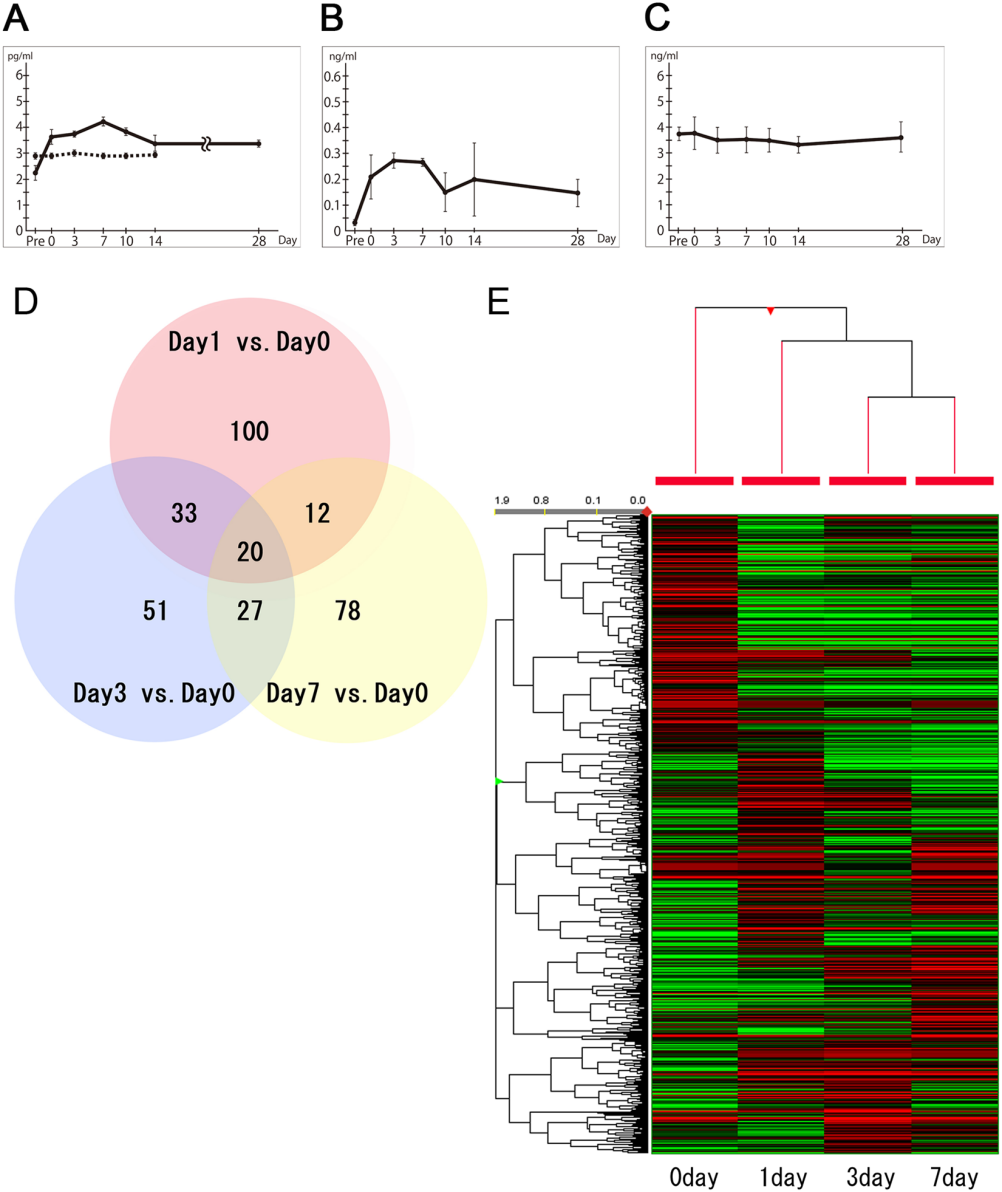
Molecular response to arterial patch grafting, as determined by enzyme-linked immunosorbent assay (ELISA) and cDNA microarray analysis. (**A**–**C**) Serum ELISA data. (**A**) In the arterial patch model (solid line), vascular endothelial growth factor A (VEGFA) levels rose rapidly immediately after the operation. They gradually increased until day 7 (solid line). A control model in which a vein patch was placed in the left common jugular vein did not exhibit any changes in serum VEGFA levels after the operation (dotted line). (**B**) Hypoxia-inducible factor (HIF)-1α responded similarly to VEGFA except that it showed a small drop on day 10. (**C**) Transforming growth factor (TGF)-β1 levels did not show any marked changes throughout the observation period. (**D**) cDNA microarray data. The Venn diagram shows the relationships between the genes that were upregulated by more than 4-fold at the graft site on days 1, 3 and 7, as compared with day 0. Twenty genes were upregulated on all 3 days. (**E**) Heat map of differentially expressed genes. Gene expression markedly changed after day 0.

Enzyme-linked immunosorbent assays (ELISAs) showed that the serum VEGFA and hypoxia-inducible factor (HIF)-1α levels increased after the patch procedure (Fig. 6A,B) whereas the transforming growth factor (TGF)-β1 levels did not change (Fig. 6C). Specifically, the VEGFA levels rose immediately after the patch procedure (day 0) and continued rising before peaking on day 7 (Fig. 6A). By contrast, the control venous patch-into-vein model did not exhibit any significant changes in serum VEGFA levels. In terms of HIF-1α level, it also rose sharply after patch surgery and peaked somewhere between days 3 and 7 (Fig. 6B).

Figure 6D shows how many genes were upregulated on days 1, 3 and 7 compared with day 0. Supplementary Table 1 lists the upregulated genes. Of the 23,364 genes that were evaluated, 165, 131 and 137 genes were upregulated by more than 4-fold on days 1, 3 and 7, respectively (Supplementary Table 1).

The cDNA microarray results for *VEGFA* and *TGF-β1* were consistent with the serum ELISA results for these proteins: compared with the day 0 expression, *VEGFA* expression on days 1 and 3 was augmented by 7.1-fold and 8.9-fold, respectively, whereas *TGF-β1* gene expression did not change. The cDNA microarray result was not available for *HIF-1a*, probably due to poor hybridization signals.

The cDNA microarray data also showed that several other angiogenesis-related genes were upregulated on days 3 and 7 relative to the day 0 expression profiles. These genes included vascular cell adhesion protein-1, fibroblast growth factor (FGF)−7 and the metalloproteinase (MMP) family. Unsupervised two-way hierarchical clustering analysis revealed that the gene expression patterns at days 1, 3 and 7 markedly differed from that at day 0 (Fig. 6E). Moreover, the gene expression patterns at days 3 and 7 differed from that at day 1. According to the Kyoto Encyclopaedia of Genes and Genomes (KEGG), the focal adhesion pathway, MAPK signalling pathway, HIF-1 signalling pathway, VEGF signalling pathway and fluid shear stress and atherosclerosis pathway were activated on days 1, 3 and 7 (Supplementary Figure 2).

We verified the cDNA microarray results by performing quantitative real-time PCR analysis of *serum amyloid A3* (*SAA3*) and *VEGFA* on days 0, 1, 3 and 7. The δCt values on days 0, 1, 3 and 7 were 6.92, −1.96, −2.99 and −0.07 for *SAA3*, respectively, and 6.56, 4.17, 0.64 and 2.96 for *VEGFA*, respectively. These results demonstrated that *SAA3* and *VEGFA* were up-regulated on days 1, 3 and 7 compared with day 0, validating the cDNA microarray data.

## Discussion

Our model of AVM/AVF formation is novel for several reasons. First, our model did not require administration of an angiogenic factor to induce neovasculature formation. By contrast, other methods of studying angiogenesis often involve adding or inhibiting one or more angiogenic factors^8–11^; for example, Wafai *et al*. showed that treatment with basic FGF or VEGFB improves the vascular deficits caused by bilateral femoral artery ligation^10^, and Penafuerte *et al*. showed that antagonising TGFβ inhibits tumour angiogenesis^10,12^. Second, we did not induce local ischemia in our patch procedure and the control models in this study show that the neovasculature in our model did not form as a result of granulation tissue formation. By contrast, many other *in vivo* angiogenesis studies have focused on wound healing, hind-limb ischaemia^10,13^ or a fibrin matrix with an arteriovenous loop^14^. Third, the neovasculature induced in our model made direct connections between pre-existing (host-origin) vessels. By contrast, the neovasculature in other studies was formed between the artery and the capillary network^13,15^. Indeed, except for a single AVM model that required genetic engineering^7^, there are no reports of a neovasculature that originates from an artery and traverses into a vein without first passing through the capillary network. Fourth, an AVF formed spontaneously in our model. Moreover, our FISH analysis showed that the neovasculature around the patch was of arterial patch (graft) origin. However, at the same time, we observed neovasculature in regions distant from the patch site that were not of graft origin. Thus, we speculate that the anastomosis resulting in the AVF involves interactions between the graft and host tissue.

Arteries and veins are considered to be quite distinct tissues since they involve different directions of blood flow and have a number of key functional differences. Moreover, arterial endothelial cells express Ephrin-B2 whereas venous endothelial cells express its receptor Eph-B4. Knockout of Eph-B4 results in defective angiogenesis of both arteries and veins. Thus, it is thought that at least some of the different properties of arteries and veins are genetically determined during ontogeny^16^. However, despite the various lines of evidence that suggest that arterial and venous endothelial cells have unique cellular signals and different functions^17^, it remains unclear how arteries and veins differ in terms of angiogenesis and vasculogenesis^18–20^. We showed in this study that exposing an arterial graft to venous blood leads to spontaneous blood vessel formation; we also showed that this angiogenesis does not occur when a venous patch is grafted into a vein or when an arterial patch is transplanted into either a vein or an artery. This in turn suggests that the differences between veins and arteries are responsible for AVM/AVF formation. While we have not yet identified which differences are responsible for the neovascular formation in our model, they may include vein-specific factors such as low blood pressure, slow flow and low oxygen concentration: one or more of these factors may have caused the artery patch to initiate vasculogenesis and angiogenesis. Thus, our model is likely to be highly useful for understanding the vein- and artery-related factors that shape vasculogenesis, angiogenesis and vascular diseases.

Our ELISA study showed that serum VEGFA and HIF-1α levels started rising immediately (*i*.*e*., within 5 min) of completing the patch procedure, which took less than an hour. In terms of VEGFA, it is unlikely that this rapid response was mediated by transcription, which involves the activation of hypoxia response element^21^. It is more likely that the surgery caused the endothelial cells to produce thrombin receptor or disrupted the homeostasis between the endothelial cells and platelets in terms of the production of nitric oxide and prostaglandins; these changes in turn may have caused the platelets, which are a known reservoir of VEGF, to release this cytokine^22–25^. In terms of HIF-1α, it is possible that the rapid response is due to changes in the oxygen levels since the levels of the HIF-α subunit are regulated by oxygen-dependent ubiquitin-proteasomal degradation, as has been described in detail elsewhere^23^.

Chu *et al*. identified 367 genes that positively regulate angiogenesis by combining the dynamic gene expression time-course data of *in vitro* VEGFA-stimulated endothelial cells with the protein-protein interactions that are known to associate with angiogenesis^26^. In the present study, we found that 167 genes are significantly upregulated in the arterial patch 1 day after transplantation compared with immediately after surgery. Of these, 12 were positive regulators identified by Chu *et al*., namely, VEGFA, BMX, C-C chemokine receptor 1 (CCR1), egl-9 family hypoxia-inducible factor 3 (EGLN3), FGF7, interleukin-1A, interleukin-1 receptor type 2, intereukin-1 receptor accessory protein, syndecan 3, MMP-9, MMP-10 and TIMP1^26^. Thus, even though there were marked differences between our and Chu *et al*.’s experimental model (*e*.*g*., we conducted an *in vivo* experiment whereas Chu *et al*. conducted *in vitro* experiments with VEGFA-stimulated endothelial cells), we identified a number of genes that were upregulated in both settings^26^. This suggests that VEGFA participated in the neovascularization observed in our model. This is supported by the rapid rise of serum VEGFA in our model rabbits and by real-time PCR and pathway analyses. In addition, hierarchical clustering analysis revealed that the global gene expression pattern was changed after arterial patch transplantation. This might be due to the simultaneous alteration of multiple pathways, including the VEGF pathway.

AVMs, along with haemangioma and telangiectasia, are human vascular diseases. While the clinical consequences of AVM (*i*.*e*., cerebral haemorrhage or subarachnoid haemorrhage) are well understood, its aetiology remains unclear. In large part, this is because it is difficult to detect AVMs in their early stage of pathogenesis, which would allow their progression over time to be monitored. The lack of a suitable model of spontaneous AVM development has also contributed to our poor understanding of this process. The new vessels that formed spontaneously in our model are very similar in terms of gross appearance and physical and histological features to the proliferating abnormal vessels in AVMs, which cannot be classified as either veins or arteries. Takagi *et al*. identified 49 genes that are up-regulated by more than 300-fold in human AVM via microarray analysis^27^. Of these, eight genes, namely, *MMP9*, *MMP12*, *PTX3*, *S100A8*, *S100A9*, *IL1R2*, *BCL2A1* and *PRG4*, were also up-regulated in our angiogenesis model. This suggests there are similarities in gene expression, in addition to morphology, between our angiogenesis model and human AVM. Thus, our model may be highly useful for elucidating the process by which human AVMs form.

In our model, the arterial wall is artificially exposed to venous blood flow. Notably, this also occurs when congenital heart diseases that result in mixed arteriovenous blood flow into the pulmonary artery are treated by surgical procedures, namely, the Glenn procedure, Fontan surgery and total cavopulmonary connection surgery^25^. These surgeries involve the anastomosis of the pulmonary artery with the vena cava^25,28,29^ and cause venous blood (which has lower oxygen saturation levels than the mixed arteriovenous blood that was flowing to the lungs before surgery) to flow directly into the pulmonary artery. Interestingly, pulmonary AVM (PAVM) is a well-known complication of these procedures^29^. Two studies suggested that hepatic factors may play a role in the development of the PAVMs after these surgeries^30,31^ because only the superior vena cava, not the inferior vena cava (which carries hepatic venous return), is anastomosed to the pulmonary artery. Thus, it has long been believed that PAVM is due to the absence or uneven distribution of hepatic venous return^32^. However, this hypothesis has been debated by other researchers^33^. Our model suggests instead that PAVM may arise after cavopulmonary anastomosis because the mixed arteriovenous blood changes suddenly to pure venous blood: this environmental change in the pulmonary artery may promote angiogenesis and PAVM formation.

Our study has several limitations. First, the effects of the low shear stress of venous blood cannot be evaluated separately from those of the low oxygen concentration using our model. Second, it is unclear if similar experiments can be performed in other mammals or using other vessels. Investigations using cultured endothelial cells, in which the levels of shear stress and oxygen can be altered independently, are required to clarify the underlying mechanisms. Additionally, experiments should be performed in other mammals or using other vessels to determine if this model is only applicable to the rabbit carotid artery and vein. Furthermore, the validity of this procedure as an animal model of AVM is uncertain. Although the neovasculature observed in this model is histologically similar to AVM, its location and macroscopic appearance are not identical to those in AVM.

In conclusion, we developed a model of spontaneous AVM/AVF in this study. It will be highly useful for vascular research, particularly in terms of the pathogenesis of various vascular diseases, including AVM.

## Materials and Methods

### Rabbits

All experiments were performed with Japanese white rabbits that weighed 3–4 kg. For most experiments, male rabbits were used. However, in the FISH experiment (see below), both male and female rabbits were used. All animal experimentation protocols were approved by the Animal Research Committee, Akita University Graduate School of Medicine (Approval number: a-1–2853). All experiments were performed in accordance with relevant guidelines and regulations.

### Generation of the rabbit model of AVM/AVF formation using the patch procedure (Akita Rabbit AVM/AVF Model)

The rabbits were pre-medicated with ketamine hydrochloride (2 mg/kg; Daiichi Sankyo Co., Ltd., Tokyo, Japan) and xylazine (3 mg/kg; Nippon Zenyaku Kogyo Co., Ltd, Fukushima, Japan). General anaesthesia was then induced using N_2_O, O_2_ and sevoflurane (Maruishi Pharmaceutical Co. Ltd, Osaka, Japan). After the animals had been sufficiently anaesthetized, a midline incision was made in the neck and the LtCCA was revealed by dissection (Fig. 1A). The blood flow in the LtCCA was measured using an ultrasonic blood flow meter (Transonic T402, Transonic Systems Inc. NY, USA). The LtCCA was then ligated: to eliminate the influence of thyroid ischaemia, the ligation occurred at a point approximately 5 mm distal of the LtCCA bifurcation of the thyroid artery (towards the head) (Fig. 1B). Arterial tissue that was 6–7 mm long was then collected. The disconnected arterial section was opened into a rectangular shape (7 × 5 mm), trimmed into an oval shape (7 mm wide × 5 mm long) and then sutured into the wall of the LtCJV using a patchwork technique with 8–0 nylon under the haemostasis of the LtCJV (Fig. 1C,D). Thereafter, the patency of the LtCJV around the arterial patch graft was confirmed using an ultrasonic blood flow meter. The LtCCA was left ligated and disconnected. After wound closure, the rabbits were housed in cages.

### Experiments with the arterial patch-bearing rabbits

Fifty-one rabbits were subjected to the patch procedure. Of these, 27 were used to assess vessel formation. Three rabbits at each time point (1, 3, 7, 10, 14, 28, 56 and 84 days and 1 year after the patch procedure) were anaesthetized and their patch-bearing LtCJV was photographed and subjected to angiography; shortly thereafter, the rabbits were euthanized and the patch-bearing LtCJV was subjected to histology. Another four rabbits that underwent the patch procedure were used to serially measure the saturated venous oxygen levels in the patched LtCJV on days 0 (5 min after the procedure), 3, 7, 10, 14 and 28. In addition, the patch-bearing LtCJV of two rabbits was excised 28 days after the patch procedure and subjected to FISH to determine whether the new vessels originated from the arterial patch. Moreover, four rabbits with the patch underwent serial vein puncture on days 0 (5 min after the procedure), 3, 7, 10, 14 and 28. Levels of VEGFA, TGF-β1, and HIF-1α in the blood were measured by ELISAs. Finally, the patches of 12 additional rabbits that had undergone the patch procedure were harvested on days 0 (5 min after the procedure), 1, 3 and 7 for cDNA microarray analysis to identify the genes that were upregulated after the patch procedure.

### Photography, angiography and histology

To observe the process of AVM formation, the rabbits were placed under general anaesthesia 1, 3, 7, 14, 28, 56 and 84 days and 1 year after the patch procedure. The neck wounds were opened, and the adhesions that had formed after the previous surgery were carefully removed so that the neovasculature could be seen. Photographs of the neovasculature were taken with a Nikon D700 (NIKON CORPORATION, Tokyo, Japan). Thereafter, to identify the new vessels and the direction of blood flow in the nascent vessels, a 6 Fr angiographic sheath (TERUMO CORPORATION, Tokyo, Japan) was inserted into the abdominal aorta and then a 4 Fr coronary angiography catheter (TERUMO CORPORATION, Tokyo, Japan) was fed into the aortic arch *via* the sheath. A surgical fluoroscope (Shimadzu Opescope Pleno, SHIMADZU CORPORATION, Tokyo, Japan) and the contrast agent amidotrizoic acid (Bayer Yakuhin, Ltd, Osaka, Japan) were used for angiography.

After taking the photographs and performing the angiography, the animals were euthanized by an injection of pentobarbital (100 mg/kg) in the ear vein. The animals were then perfused with a 4% paraformaldehyde solution in distilled water *via* a catheter that was inserted in the abdominal aorta. The blood vessels in the neck and the surrounding adipose tissue were dissected and harvested. After immersion and fixation in formalin, the LtCJV was opened from the back, namely, opposite the LtCCA graft, and the graft was observed from the luminal side with a stereoscopic microscope (SZX7, Olympus Corporation, Tokyo, Japan). The LtCJV and its surrounding tissues were then cut into 5 mm-thick slices, which were embedded in paraffin. The paraffin-embedded samples were subsequently cut into 4 μm-thick sections and stained with Haematoxylin-Eosin (HE) and Elastica-Masson (EM).

### Measurement of venous oxygen saturation

To determine when the shunt blood flow was established by the anastomosis of new blood vessels, the oxygen saturation in the LtCJV was serially measured on days 0 (within 5 min after grafting), 3, 7, 10, 14 and 28 after the patch procedure. Thus, under general anaesthesia, the neck wound was incised again, the LtCJV was exposed and the oxygen saturation of the vein was measured by placing a Nonin 9847 v pulse-oximeter (Nonin Medical Inc., Minnesota, USA) on the LtCJV slightly proximal of the LtCCA graft.

### Fluorescence *in situ* hybridization (FISH)

To analyse neovascularization, two female rabbits underwent the patch procedure with LtCCA patches obtained from male rabbits. The female rabbits were euthanized 28 days later, and their arterial patch was subjected to FISH with a Cy3-labelled rabbit Y chromosome-specific FISH probe (Chromosome Science Lab, Sapporo, Japan). Thus, formalin-fixed paraffin sections of the patch-bearing LtCJV were deparaffinized in xylene, activated by incubation in 0.1% pepsin/0.1 N HCl for 30 min at 37 °C, neutralized in PBS, washed in distilled water and dried with air. A drop of the rabbit Y chromosome-specific FISH probe was then added to the sections, and the sections were heated to 90 °C for 10 min. The sections were then incubated overnight at 37 °C. A fluorescence microscope (Model BZ-9000, Keyence Corp., Osaka, Japan) was used to observe the slides and obtain images. A Leica CW-4000 imaging workstation (Leica Microsystems GmbH, Wetzlar, Germany) was used for the image analyses.

### Enzyme-linked immunosorbent assay (ELISA)

Blood samples were collected from the right common jugular vein (RtCJV) before and 0 (5 min after surgery), 3, 7, 10, 14 and 28 days after three rabbits had undergone grafting. Plasma was obtained by transferring the blood sample into a tube containing sodium citrate anticoagulant and centrifuging it at 3000 rpm for 5 min at 4 °C. The plasma was then stored at −80 °C. The VEGFA and TGF-β1 levels in the samples were determined using a sandwich ELISA purchased from Cusabio Biotech Co., Ltd. (Wuhan, China). The HIF-1α levels in the samples were determined using rabbit HIF-1α ELISA kits obtained from LifeSpan Biosciences, Inc. (Seattle, USA). The concentration of each cytokine in each sample was calculated on the basis of the recombinant protein-based standard curve. The Multiskan JX spectrophotometer (Thermo Fisher Scientific Inc., Yokohama, Japan) was used to measure the absorbance. All assays were performed in duplicate, and the mean value of the results was used in the analyses.

### cDNA microarray analysis

Tissue samples from the arterial patch were snap-frozen in liquid nitrogen immediately (day 0) and 1, 3 and 7 days after the procedure, and total RNA was extracted using NucleoSpin® RNA (MACHEREY-NAGEL GmbH & Co. KG, Düren, Germany). Biotinylated cDNA was prepared from 100 ng of total RNA using a GeneChip WT PLUS Reagent Kit (Affymetrix, Inc., Santa Clara, CA) according to the manufacturer’s instructions. After fragmentation, 5.5 µg of single-stranded cDNA was hybridized to a Rabbit Gene 1.0 ST Array for 16 hr at 45 °C. The arrays were then washed and stained in the GeneChip Fluidics Station 450 (Affymetrix), after which they were scanned using GeneChip Scanner 3000 7 G. The data were analysed using Affymetrix Expression Console Software 1.4.1, specifically the Affymetrix Transcriptome Analysis Console (TAC) software, which offers RMA-Sketch for gene and exon level analysis. The differentially expressed genes were identified on the basis of the fold changes. The genes were considered to be upregulated when their fold change was ≥4.0.

Unsupervised two-way hierarchical clustering analysis was performed using microarray data from days 0, 1, 3 and 7. A heat map was drawn using GeneSpring version 14.8 (Agilent Technologies Japan Ltd.). To investigate the molecular pathways activated on day 1, 3 or 7 compared with day 0, pathway analysis of the microarray data was conducted with the KEGG database (http://www.kegg.jp/kegg/kegg1.html)^34^ and an in-house script. All significantly up-regulated genes on day 1, 3 or 7 were investigated and annotated with biological processes, protein-protein interactions and gene regulatory networks using the KEGG database^35^. All identified pathways were mapped individually.

### Quantitative real-time PCR

Expression of *SAA3* (Oc03397898_m1) and *VEGF* (Oc03395999_m1) was evaluated by TaqMan quantitative real-time PCR assays (Applied Biosystems, Life Technologies, Grand Island, NY, USA) using RNA extracted from freshly frozen tissues. *β-Actin* (Oc03824857_g1) was used as the endogenous control to normalize target gene expression. Quantitative real-time PCR was performed on a 7900HT Fast Real-Time PCR System (Applied Biosystems) and data were collected and analyzed using SDS 2.3 software. All assays were performed in triplicate. Gene expression was quantified by calculating δCt values, where Ct = threshold cycle and δCt = Ct of target gene − Ct of *β-Actin*. DataAssist 2.0 (Applied Biosystems) was used to analyze changes in expression.

### Surgical control and other experimental rabbit models

Three rabbit models that served as controls for various aspects of the patch procedure were created surgically. Three rabbits were used to generate each model, and all animals were subjected to histology as described above 28 days after surgery. These models were as follows:

1. Incision model: To account for the possibility that granulation tissue created by surgery induced the new vessel formation, the LtCCA and LtCJV were subjected to the same incisions used in the patch procedure. However, graft implantation was not performed. Similar to the patch procedure, the incised vessels were sutured using 8–0 nylon sutures (Supplementary Figure 1A).
2. Carotid artery ligation model: This model was designed to confirm that ischaemia in the surrounding tissue caused by ligation of the carotid artery did not promote angiogenesis. Thus, the neck was incised and the LtCCA was ligated in two places approximately 1.5 mm apart. The neck wound was then closed (Supplementary Figure 1B).
3. Carotid artery dissection model: Whether LtCCA dissection participated in the new vessel formation was evaluated by making an incision in the neck and ligating and dissecting the LtCCA. The LtCCA was left ligated and disconnected. After making an incision in the LtCJV and then suturing it with 8–0 nylon sutures, the neck wounds were closed (Supplementary Figure 1C).

Another three models (three rabbits per model) were created to examine the effect of changing the patch source vessel and/or the recipient vessel on new angiogenesis. All were subjected to histology 28 days after surgery. The first model, namely, the venous patch-into-vein model, was also subjected to ELISA to measure the serum VEGF levels before and immediately (day 0) and 3, 7, 10 and 14 days after surgery. This venous patch-into-vein model was generated to assess whether angiogenesis also occurs when the patch is from another vein rather than from an artery. Thus, the right external jugular vein was ligated and harvested: the resulting venous tissue patch was sewn into the LtCJV in the same manner as in the patch procedure using 8–0 nylon thread (Supplementary Figure 1D). The next model was a venous patch-into-artery model: it was generated to assess whether angiogenesis also occurs when the patch is from a vein and it is placed in an artery. Thus, the same right external jugular vein-derived patch used in the venous patch-into-vein model was sutured into the LtCCA (Supplementary Figure 1E). Finally, the arterial patch-into-artery model was designed to assess whether angiogenesis also occurs when an arterial patch is placed in an artery. Thus, arterial tissue harvested from the right common carotid artery (RtCCA) was transplanted into the LtCCA (Supplementary Figure 1F).

## Electronic supplementary material

Supplementary Information

Video 1 Angiography Day28

Video 2 Angiography 3months
