# Supplementary material for: Neovasculature can be induced by patching an arterial graft into a vein: A novel in vivo model of spontaneous arteriovenous fistula formation
Source: Sci Rep. 2018 Feb 16;8:3156. doi: 10.1038/s41598-018-21535-2 (PMC5816615; doi:10.1038/s41598-018-21535-2)
Supplement: Supplementary file 1 — Supplementary Information [file 41598_2018_21535_MOESM1_ESM.docx]

*Original Article*

**Neovasculature can be induced by patching an arterial graft into a vein: A novel *in vivo* model of spontaneous arteriovenous fistula formation.**

Yukinobu Ito^1)^, Makoto Yoshida^1)^, Daichi Maeda^1)^, Masato Takahashi^2)^, Hiroshi Nanjo^3)^, Hirotake Masuda^4)^, Akiteru Goto^1)^ *

1. Department of Cellular and Organ Pathology, Graduate School of Medicine, Akita University, Akita, Japan.
2. Department of Diagnostic Pathology, Akita Kousei Medical Center, Akita, Japan.
3. Department of Clinical Pathology, Akita University Hospital, Akita, Japan.
4. Department of Clinical Laboratory, Ogachi Central Hospital, Akita, Japan.

*Corresponding author:

Akiteru Goto, MD, PhD

Department of Cellular and Organ Pathology, Graduate School of Medicine, Akita University,

1-1-1 Hondo, Akita, Akita 010-8543, Japan

E-mail: akigoto@med.akita-u.ac.jp

Phone: 81-18-884-6062

Fax: 81-18-884-6441

**Keywords**: angiogenesis, animal model, arterial patch graft, arteriovenous malformation, arteriovenous fistula, enzyme-linked immunosorbent assay (ELISA), cDNA microarray

**Supplementary Figure 1 Surgical control models and other experimental models.** Several additional models were created surgically. The left common carotid artery (LtCCA) is shown in red, and the left common jugular vein (LtCJV) is shown in blue. (A) Incision model: the LtCCA and the LtCJV were incised and then sutured with 8-0 sutures. (B) Ligation model: the LtCCA was ligated only. (C) Dissection model: a 10 mm section of the LtCCA was excised. (D) Venous patch-into-vein model: the venous patch was harvested from the right external jugular vein (light blue patch) and sewn into the LtCJV. (E) Venous patch-into-artery model: a venous patch (blue patch) was harvested from the right common jugular vein and sewn into the LtCCA. (F) Arterial patch-into-artery model: an arterial patch was harvested from the right common carotid artery (red patch) and sewn into the LtCCA.

**Supplementary Figure 2.** Maps of the (A) focal adhesion signalling pathway, (B) MAPK signalling pathway, (C) HIF-1 signalling pathway, (D) PI3K-Akt signalling pathway, (E) VEGF signalling pathway and (F) fluid shear stress and atherosclerosis pathway based on the latest Kyoto Encyclopaedia of Genes and Genomes (KEGG) database. Yellow nodes are up-regulated and green nodes are not significantly changed.

Supplementary Table 1. The genes that were upregulated at the graft site on days 1, 3 and 7, as compared with on day 0

Supplementary Figure 1


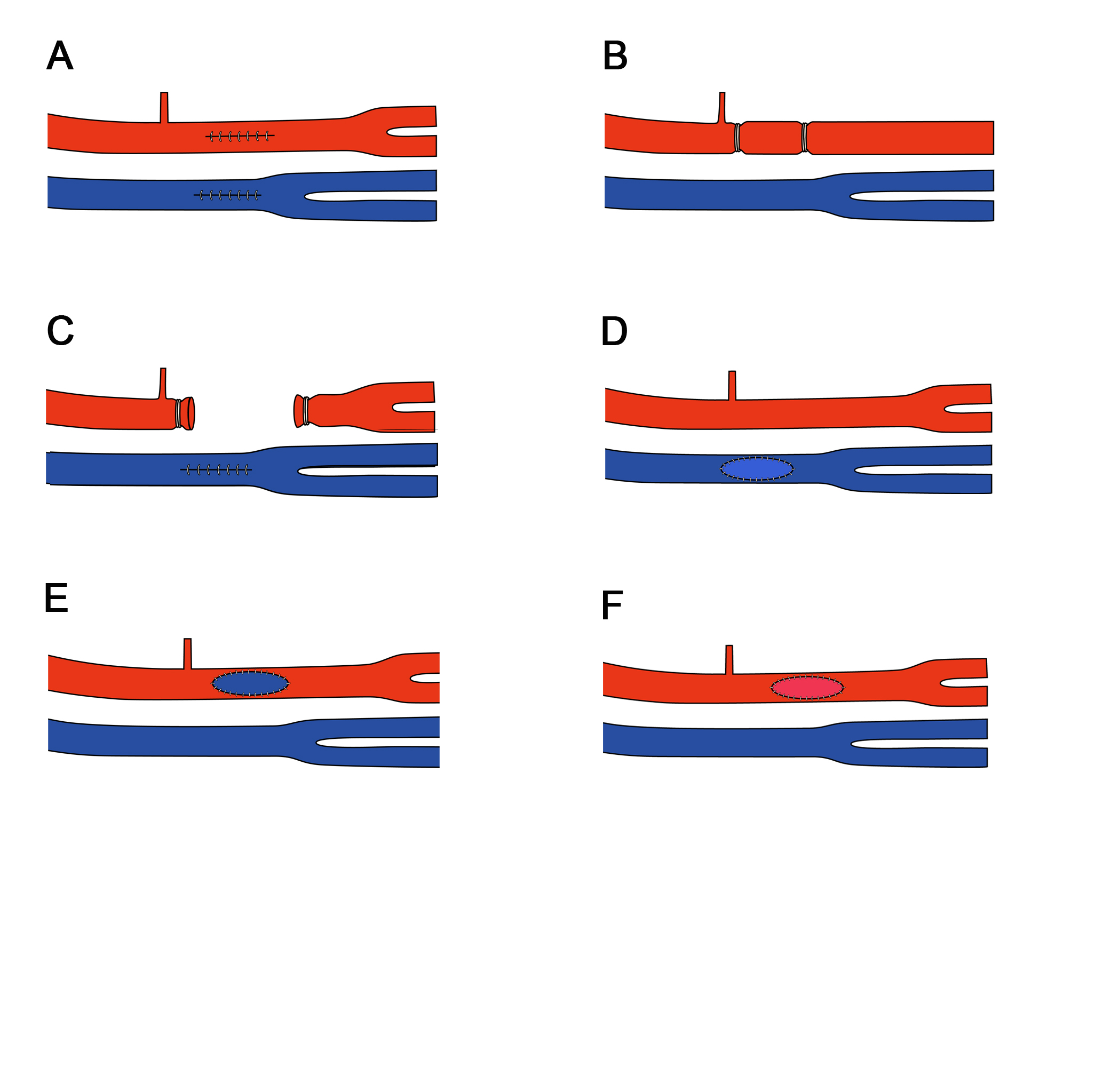


Supplementary Figure 2-A


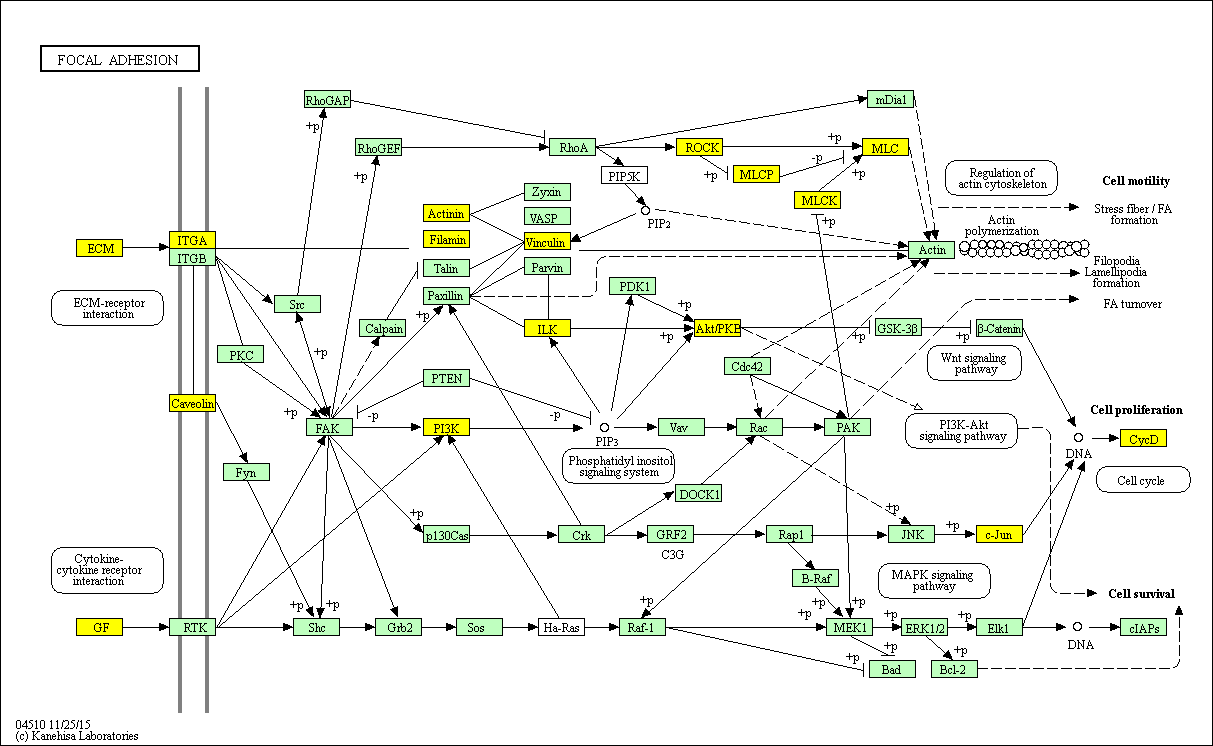


Supplementary Figure 2-B


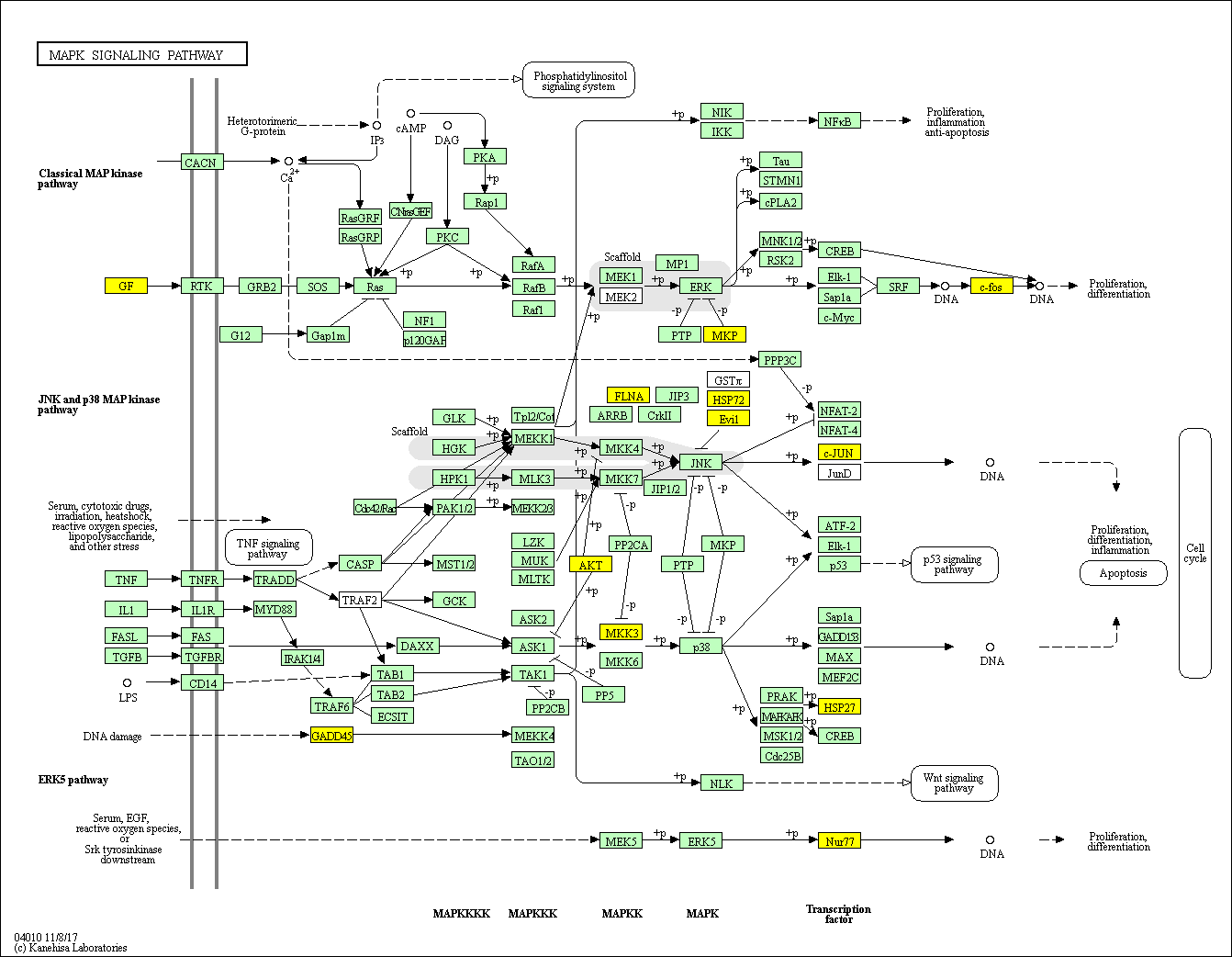


Supplementary Figure 2-C


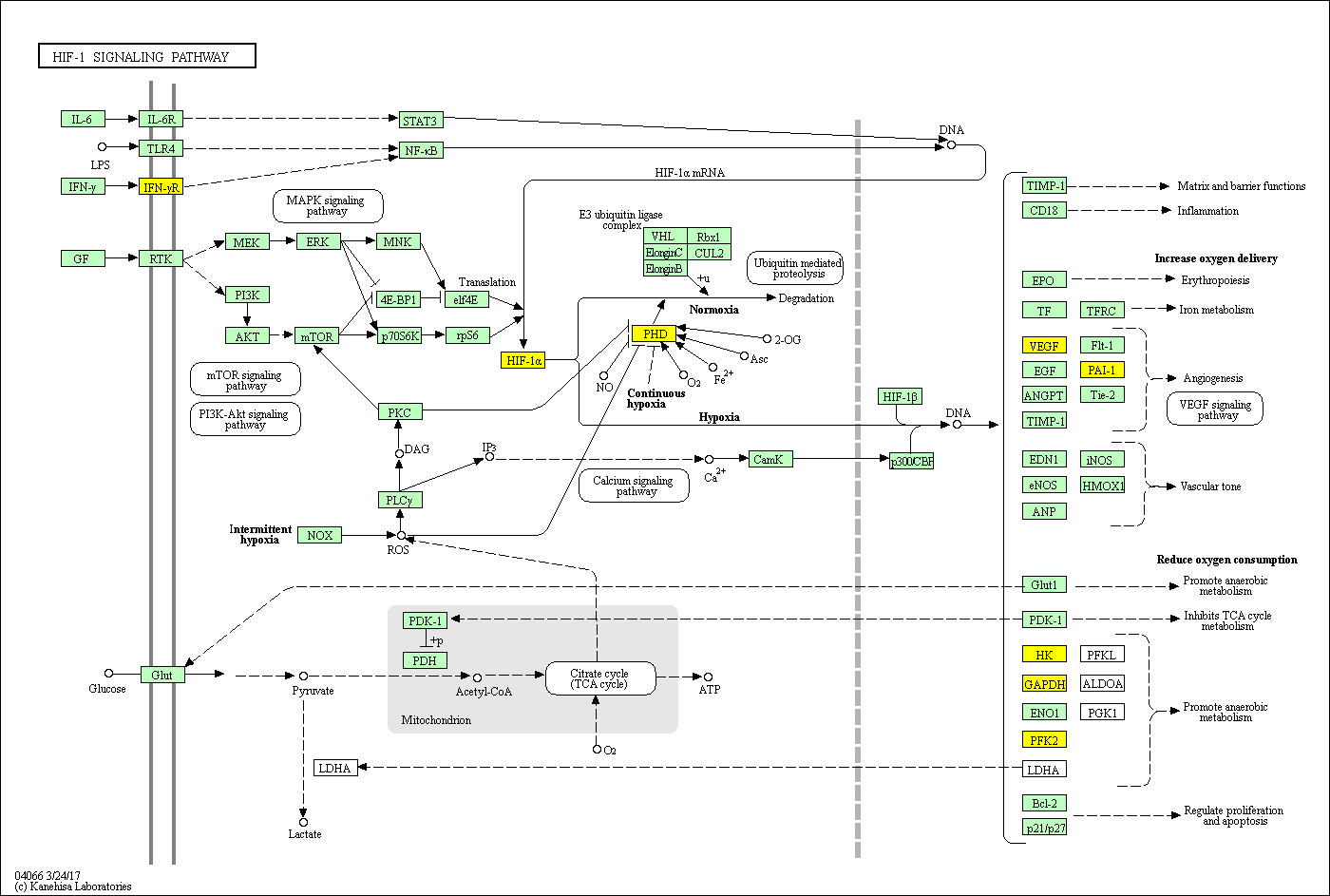


Supplementary Figure 2-D


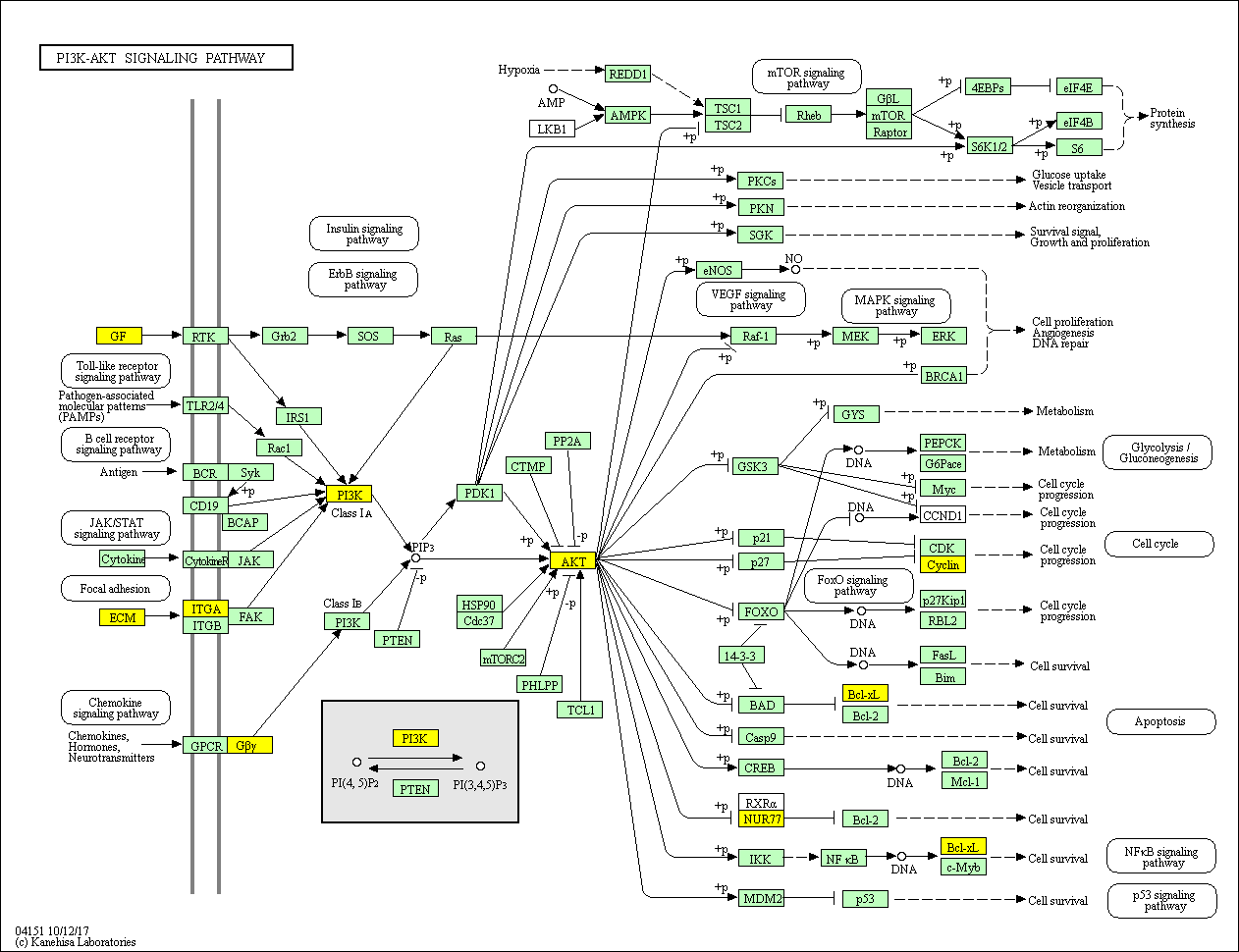


Supplementary Figure 2-E


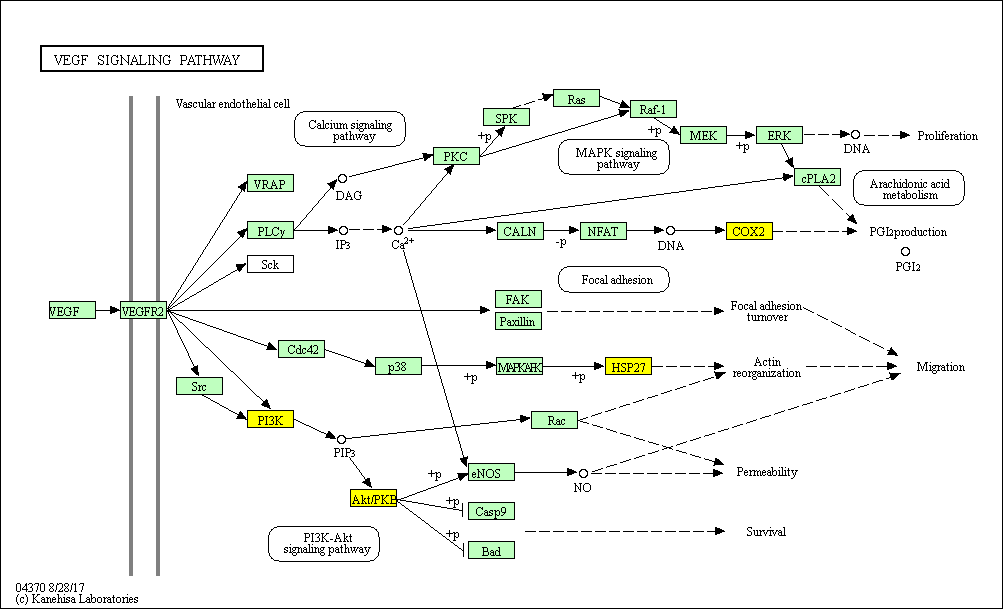


Supplementary Figure 2-F


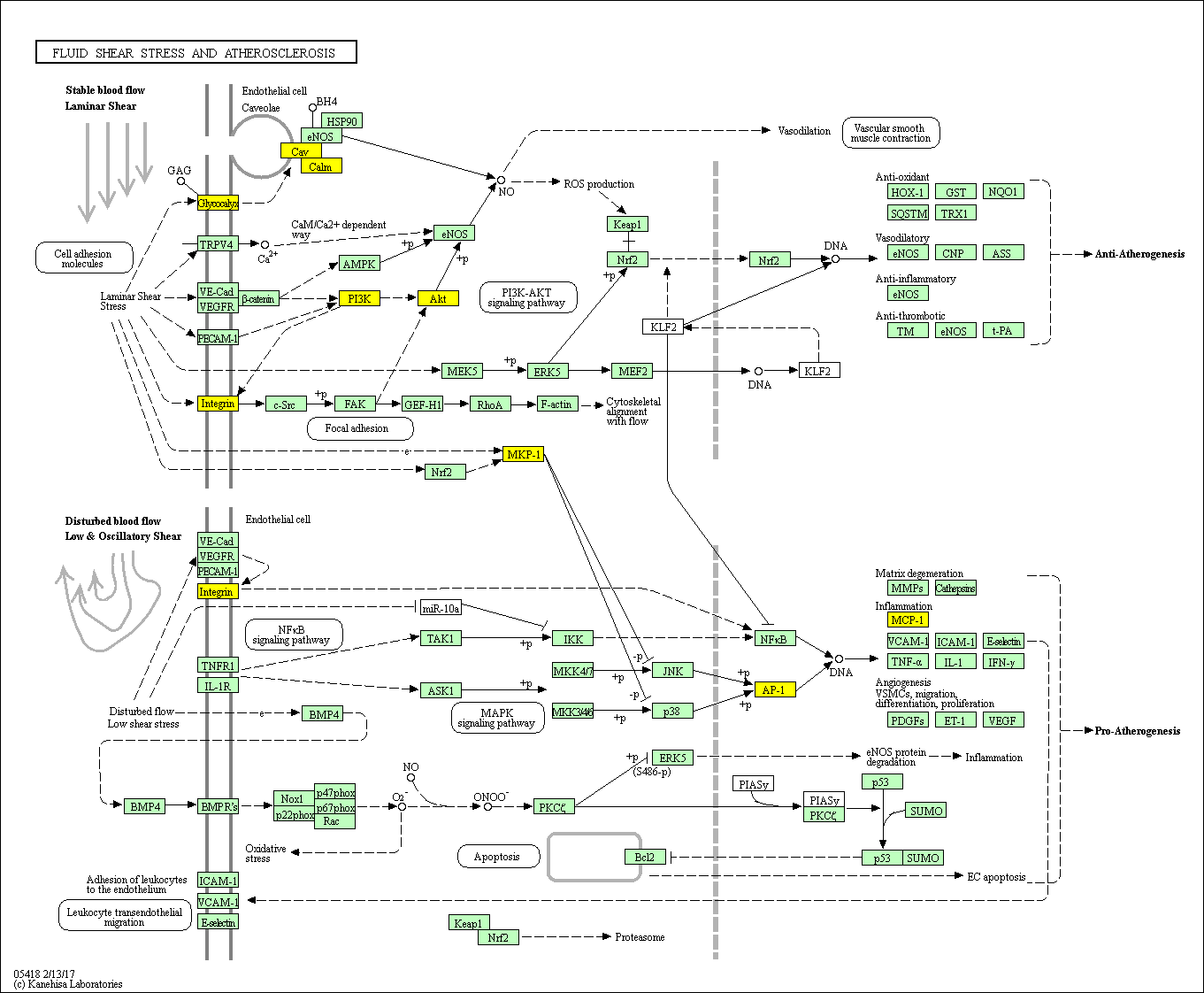


Supplementary Table 1.
